# Effective communication during disease outbreaks: the role of data journalism in pandemic and epidemic intelligence

**DOI:** 10.1186/s12919-025-00319-3

**Published:** 2025-03-04

**Authors:** Barbara Tornimbene, Zoila Beatriz Leiva Rioja, Aghnia Adzkia, Christian Endt, Rukmini S., Oliver Morgan

**Affiliations:** 1 World Health Organization Hub for Pandemic and Epidemic Intelligence, Berlin, Germany; 2 CPC Analytics, Berlin, Germany; 3 BBC World Service, Jakarta, Indonesia; 4 Die Zeit, Berlin, Germany; 5 Independent Data Journalist, Delhi, India

**Keywords:** Data journalism, Science communication, Pandemic intelligence, Public health collaboration, Misinformation management

## Abstract

The COVID-19 pandemic highlighted the importance of journalism, especially data journalism, in conveying accurate and understandable scientific information. Journalists helped to convert difficult scientific findings into understandable narratives, improving public understanding and trust. During the fifth session of the WHO Pandemic and Epidemic Intelligence Innovation Forum, data journalists Rukmini S. (India), Christian Endt (Germany), and Aghnia Adzkia (Indonesia) discussed their pandemic reporting experiences. The collaboration among media, public health agencies, and academia was critical in guaranteeing fast and accurate data transmission. During the session, they also discussed the obstacles journalists confront, such as overcoming data gaps and resolving public mistrust caused by misinformation or imprecise government messaging. In response, journalists' aim is to bridge the communication gap between scientists and the general people, ensuring that even complex and unclear scientific findings could be understood. Moving forward, the emphasis is on the ongoing collaboration between data journalists, scientists, decision-makers and the public to improve knowledge and science communication. Data journalism will continue to be important in future public health emergencies because it promotes transparency, makes data available, and encourages public engagement. Collaborative efforts, technical briefings, and training opportunities will improve data journalists' ability to effectively report scientific breakthroughs, making public health communication more responsive and impactful.

## Introduction

The COVID-19 pandemic caused considerable disruption to the way we lived and worked, affecting almost every aspect of our lives [1]. During the pandemic, journalists played an important role in providing information and insights to members of the public [2]. With the widespread availability of information, journalists have developed a considerable ability to translate complex scientific information into a form that is accessible to their readers [3, 4]. In addition to translating scientific information, journalists also acted as trusted sources of information about the pandemic, ensuring accurate reporting and interpretation of information. Journalism itself has an important role during public health outbreaks that includes providing balanced coverage of news and helping to create a more informed and engaged public, which ultimately contributes to the adoption of measures for disease control [5].

During the sixth World Health Organization (WHO) Pandemic and Epidemic Intelligence Innovation Forum [6], convened in two sessions, one on 9th March and the second on 14th April 2023, three data journalists who were highly involved in reporting during the COVID-19 pandemic, shared their experiences: Rukmini S., an independent data journalist based in Chennai, India, who has worked for several very important media outlets in her country; Christian Endt, representing Zeit Online, who works as senior data journalist; and Aghnia Adzkia, senior visual journalist for the East Asia Hub of the BBC World Service based in Indonesia. The meeting provided an opportunity to discuss the credibility and trust in the context of science communication. The discussion also touched on how journalists struggled with information gaps and the complexity of the science during the pandemic. Finally, participants discussed the importance of open collaboration among public institutions, academia, and media outlets in sharing knowledge and resources in the field of science communication. This article aims to summarize the key insights from the discussion while exploring their implications for strengthening science communication and collaboration in future public health crises.

## Why data journalism mattered during the pandemic and beyond

Data journalism is a key component of science communication [7, 8]. It became increasingly important in light of the COVID-19 pandemic and its associated changes [2]. With the rise of online communication and the greater availability of information, data journalism was essential in helping communicate the truth and provide context to the data surrounding the pandemic[7]. All presenters were involved with the creation of COVID-19 dashboards and visualizations tools to document the virus's spread, giving people access to information that was frequently not available through traditional news outlets. The BBC website with maps of COVID-19 spread was translated in different language and its East Asia Hub created national maps for the countries in the region. They also gave light to local and regional stories, which often did not receive the attention at the international level. They replicated the every-day story of a girl on lockdown in Wuhan and documented real-life stories of people that died due to COVID-19 in Indonesia. In the case of India, reporting the number of deaths did not just support high-level decision making but conveyed a sense of closure and dignity, even in death, to people who at that moment felt robbed of it. Numbers have the power to give people closure in difficult times.

All presenters stressed the crucial role played by data journalists in helping scientists interact with the public and gathering their feedback. By supporting scientists and the public authorities in being open and honest about methodologies, assumptions, and uncertainty associated with them, data journalism helps fulfill multiple needs [9]. When it comes to transparency, privacy, anonymity, and access control, partnership among the health authorities and academia, journalists can help to stimulate the interaction of all the involved actors and break down obstacles that in the past have limited effective communication [10, 11]. In the example of Germany, data journalists also helped to prevent the spread of misinformation and disinformation, which was a serious concern during the pandemic. In times of crisis, the accuracy of scientific data and the clarity of communication are critical and when these are lacking, it can lead to confusion and sometimes resistance from both members of the public and governments.

Data journalism has evolved into a potent instrument for enhancing public understanding of the world and scientific developments. It also plays a crucial role in aiding scientists and public authorities in formulating effective disease control strategies. During the session, it was emphasized that data journalists should further enhance their grasp of scientific data and communicate it in a manner that fosters trust within communities. Simultaneously, they must adhere to a rigorous code of conduct, encompassing quality control measures in newsrooms, where every article undergoes verification and fact-checking prior to publication [12, 13].

## Communicating science credibly and building trust with the public

Science inherently lacks perfection and definitiveness; it operates as a hypothesis-driven endeavour, with scientists continuously refining their knowledge as more data becomes available. This dynamic process becomes especially pronounced during crises, such as a pandemic, when data may be incomplete. Unfortunately, there is often a political expectation to present information as definitive to build public trust, yet the inherent uncertainty in science can lead to public mistrust. During the COVID-19 pandemic in India, public trust eroded due to the government's approach to sharing information, and in Germany, a similar tension between politicians and scientists emerged [14, 15].

An example of the dissonance between how science works and how the public believes it works was reflected in the way citizens in India reacted to COVID-19 mortality and estimates of excess mortality published by the government. Accurate estimates of mortality are key to understanding and responding to a pandemic, but the data used to calculate them must be as accurate and up to date as possible. However, even countries with quite sophisticated statistical systems were not able to gather and report information quickly enough to generate those metrics. Additionally, the lack of resources and surveillance capacities contributed to reporting artefacts that made it difficult to obtain a clear understanding of the situation. Furthermore, there may have been other incentives for some states to under-report the situation, such as political pressure to present a more favourable picture or to deflect criticism and secure funding. Overall, limited data availability impacted on the accuracy of the calculations and often published data did not reflect local realities. In some instances, this led to communities’ Civil society groups collecting and reporting their own death data to ensure that they were represented in overall statistics. This apparent data gap created a sense of mistrust in the population and the idea that scientists and/or government were hiding real information, fuelling conspiracy theories and confusion among the public. In Germany, there was a lot of discussion about the efficacy and risk of COVID-19 vaccines, and this was used to further polarize an already divided and sceptical audience [16, 17]. Poor data availability, slow reporting capacity at national level, lack of modelling skills for epidemics, and lack of transparency when it came to the limitations of science, were some of the challenges to the perceived credibility of public authorities and academia.

During the forum's open discussion participants discussed factors that can lead the audience to lose trust in science and how this is also linked to the level of complexity of the information that is communicated. For example, it was mentioned how sometimes the interests of the science, and the public do not align. This can create a conflict between ensuring the public receives timely, accurate information and the inherent complexity and evolving nature of scientific findings. While scientists may feel pressure to publish quickly to address urgent public needs or to claim priority in their field, such rushed publications may lack sufficient validation, increasing the risk of inaccuracies and eroding public trust when initial findings are later revised. Additionally, the complexity of scientific information can make it difficult to convey the nuances to a general audience without risking misinterpretation. In general, in the context of an epidemic, scientists produce data with the primary focus of sharing them with public institutions to support decision-making, so it is often necessary to sacrifice the time needed to make the science more understandable to protect the public's trust in favour of providing accurate information quickly. This can lead to the public ether misunderstanding or simply rejecting the information because of its complexity, to the point the public might feel that something is hidden from them, or that changes in scientific opinions are not clearly justified.

All presenters agreed that ultimately, the choice should always be to share available information as soon as possible, even if not completely accurate or usually complex, and that transparency becomes the key when it comes to building trust. Ultimately, it is the role of data journalists to bridge the communication gap between scientists and the public, making scientific information more accessible and ensuring it is presented in a way that avoids misinterpretation or unnecessary panic. This includes contextualizing reports, such as the emergence of a new variant, to prevent overreactions like border closures or flight cancellations, while still conveying the seriousness of the situation accurately.

## Collaboration for successful science communication

The COVID-19 pandemic has provided an opportunity for data and evidence to be shared more widely than ever before. This has been made possible through increased collaboration between journalists, health authorities, and academia, as well as the sharing of knowledge of data access and methodologies between journalists. In the case of BBC Indonesia this collaboration has helped lighten the burden on public health authorities, who were often overwhelmed by the need to rapidly share huge amounts of data and information. Working with journalists form different parts of East Asia, the BBC was able to deliver correct information to a variety of countries in the region, particularly when the mainstream media was focusing on Europe and North America.

The collaboration between public health authorities, journalists and academia has been particularly beneficial in bringing evidence-based stories into the public discourse and should be fostered. However, there are some aspects that need to be strengthened for better collaboration. In Germany, the lack of COVID-19 data accessibility was an issue for the work of data journalists, both in terms of nature and format. It’s important that governments supply metadata and clearly describe the methodologies used to calculate statistics on mortality, recoveries, testing, and contact tracing systems. During the discussion, it was noted that policymakers, politicians, and activists often incorrectly compared mortality data from different countries to support specific policies or political arguments, without acknowledging that these figures were frequently calculated using differing methodologies or baselines. Clarifying these differences is essential to avoid misinterpretation and ensure informed decision-making. Having access to a broader range of data types, such as economic indicators, public health statistics, mental health data, and education data on the impacts of school closures, is crucial for data journalists to provide a comprehensive picture of the situation. These data types are often siloed across different sectors, making it challenging for journalists to access and integrate them effectively. Breaking down these silos and enabling cross-sector data sharing would empower data journalists to offer deeper insights into the multifaceted impacts of events like pandemics, helping the public and policymakers to better understand and address complex crises.

Similarly, in India data deficits and lags in the reporting of official statistics made it difficult for data journalists to gather complete and accurate information to share with the public. Journalists had to rely on non-traditional approaches in data gathering and reporting to access the information they needed. Subnational efforts played a key role in this process, even when they were low-tech. Collaborating with people involved in volunteer-driven activities became an important part of this, as it helped obtaining data from communities that could not be reached by routine systems.

## Going forward and important next steps

In the wake of the COVID-19 pandemic, it is now more important than ever to invest in science communication. The importance of uncertainty and hypothesis-driven research, and the evidence-based approaches we use to solve problems should be emphasised. Data-driven conversations must be used to discuss and address public issues, as well as galvanize funding to ensure that the public is informed and understands the scientific process and its importance in their lives.

The session highlighted the growing importance of data science as a component of science journalism. Going forward, it will be helpful to create collaborative environments that promote ongoing exchanges between scientists and journalists, so that they can learn from each other and work together to strengthen science communication. Having such a collaborative environment would also make it easier for journalists to understand the scientific nuances and complexities of the data while helping scientists to communicate the results of their research in a more succinct and accessible manner.

Building an ongoing collaborative environment to further develop data journalism will require focused effort and resources. Some options could include holding regular technical briefings from leading specialists in topics such as infectious diseases modelers, as well as other technical training opportunities in data analytics for data journalists. Future directions for data journalism include many exciting opportunities, such as providing more interactive ways for members of the public to engage with data, as well as working with citizen science approaches to fill data gaps and triangulate information with communities themselves. Greater collaboration between data journalists and public health scientists provides new opportunities for better science communication, greater community engagement, and ultimately more responsive and impactful public health interventions.

## Data Availability

Not applicable.

## Abbreviations

WHO: World Health Organization
BBC: British Broadcasting Corporation
COVID-19: Coronavirus Disease 2019
EVD: Ebola Virus Disease
UKHSA: United Kingdom Health Security Agency
